# Inflammatory Cytokines and Survival Factors from Serum Modulate Tweak-Induced Apoptosis in PC-3 Prostate Cancer Cells

**DOI:** 10.1371/journal.pone.0047440

**Published:** 2012-10-15

**Authors:** Ana Belen Sanz, Maria Dolores Sanchez-Niño, Susana Carrasco, Felix Manzarbeitia, Olga Ruiz-Andres, Rafael Selgas, Marta Ruiz-Ortega, Carmen Gonzalez-Enguita, Jesus Egido, Alberto Ortiz

**Affiliations:** 1 Nefrologia, IdiPAZ, Madrid, Spain; 2 Nefrologia, IIS-Fundación Jiménez Díaz, Universidad Autónoma de Madrid and Fundación Renal Iñigo Alvarez de Toledo, Madrid, Spain; 3 Anatomia Patologica, IIS-Fundación Jiménez Díaz, Universidad Autónoma de Madrid, Madrid, Spain; 4 Urologia, IIS-Fundación Jiménez Díaz, Universidad Autónoma de Madrid, Madrid, Spain; Universitat de Lleida - IRBLLEIDA, Spain

## Abstract

Tumor necrosis factor-like weak inducer of apoptosis (TWEAK, TNFSF12) is a member of the tumor necrosis factor superfamily. TWEAK activates the Fn14 receptor, and may regulate cell death, survival and proliferation in tumor cells. However, there is little information on the function and regulation of this system in prostate cancer. Fn14 expression and TWEAK actions were studied in two human prostate cancer cell lines, the androgen-independent PC-3 cell line and androgen-sensitive LNCaP cells. Additionally, the expression of Fn14 was analyzed in human biopsies of prostate cancer. Fn14 expression is increased in histological sections of human prostate adenocarcinoma. Both prostate cancer cell lines express constitutively Fn14, but, the androgen-independent cell line PC-3 showed higher levels of Fn14 that the LNCaP cells. Fn14 expression was up-regulated in PC-3 human prostate cancer cells in presence of inflammatory cytokines (TNFα/IFNγ) as well as in presence of bovine fetal serum. TWEAK induced apoptotic cell death in PC-3 cells, but not in LNCaP cells. Moreover, in PC-3 cells, co-stimulation with TNFα/IFNγ/TWEAK induced a higher rate of apoptosis. However, TWEAK or TWEAK/TNFα/IFNγ did not induce apoptosis in presence of bovine fetal serum. TWEAK induced cell death through activation of the Fn14 receptor. Apoptosis was associated with activation of caspase-3, release of mitochondrial cytochrome C and an increased Bax/BclxL ratio. TWEAK/Fn14 pathway activation promotes apoptosis in androgen-independent PC-3 cells under certain culture conditions. Further characterization of the therapeutic target potential of TWEAK/Fn14 for human prostate cancer is warranted.

## Introduction

Prostate cancer is the second leading cause of cancer-related death in males [1]. Most cases of prostate cancer present as localized disease and may be cured with surgery and radiation. However, as is true with most solid malignancies, the development of metastatic disease is ultimately lethal. Despite active systemic therapies, the metastatic phenotype will drive in the development of resistance and disease progression. Moreover, systemic treatments in prostate cancer are limited. Until recently, there were only three FDA-approved chemotherapeutic agents for use in castrate-resistant prostate cancer (estramustine, mitoxantrone, and docetaxel) and two additional agents were approved in 2010 (sipuleucel-T and cabazitaxel [2]. However, there is still a clear need to develop additional systemic therapies. The growth of normal prostate epithelial cells is under the tight control of various growth factors, most notably androgens, castration leads to apoptosis of this cell population. Androgen-depletion has a similar effect on prostate cancers. However, following initial regression tumors often return in an androgen-depletion independent form that is frequently lethal. Thus, it is of particular interest to search for agents able to kill androgen-independent prostate cancer cells.

Tumor necrosis factor (TNF) was originally described as a factor toxic for tumors [3], [4]. It was later shown to belong to the TNF superfamily (TNFSF) of cytokines [5], [6]. Many TNFSF cytokines regulate cell death and proliferation, as well as inflammation and may have a role in tumor biology, including prostate cancer biology [7]–[9]. As an example, recent attention has focused on the anti-tumor activity of TNF-related apoptosis-inducing ligand (TRAIL) [10], [11]. However, in vivo prostate cancers express osteoprotegerin, a decoy receptor for both TRAIL and activator of nuclear factor-κB ligand (RANKL) [12]. TNFSF cytokines activate a family of receptors (TNFRSF) many of which carry a death domain (DD) and function as death receptors. Activation of death receptors in tumor cells by cytotoxic immune cells is the main mechanism by which the immune system eliminates malignant cells [13].

Tumor necrosis factor-like weak inducer of apoptosis (TWEAK, Apo3L, TNFSF12) is one of the most recent members of TNFSF to be identified [14], [15]. TWEAK was originally described as an inducer of apoptosis in tumor cells. In addition, TWEAK can regulate cell proliferation, cell death, cell migration, cell differentiation, tissue regeneration, neoangiogenesis and inflammation [16]–[24]. TWEAK activates a single receptor, fibroblast growth factor-inducible-14 (Fn14, TWEAK receptor, TNFRSF12A, CD266). TWEAK activation of the Fn14 receptor results in apoptotic cell death of multiple tumor cell lines [22], [25]–[29]. Indeed, a phase I clinical trial of a humanized anti-TWEAK receptor monoclonal antibody in subjects with advanced solid tumors was recently completed [30]. However, TWEAK-Fn14 up-regulates VEGF expression to foster ovarian cancer cell metastasis [31] and promotes breast cancer cell invasive capacity [32]. There is evidence that the different, even opposed, actions of TWEAK could be determined by the microenvironment. In this regard, TWEAK induces apoptosis in renal tubular cells in a pro-inflammatory environment, while, it promotes proliferation in presence of bovine fetal serum [33], [34]. Prostate cancer cells have been shown to express Fn14 and high expression of Fn14 was significantly associated with higher prostate-specific antigen recurrence rate in patients who underwent radical prostatectomy [35]. Fn14 was highly expressed in androgen-independent prostate cancer cell lines, DU145 and PC-3, whereas expression was weak in androgen-sensitive LNCaP cells. A role for Fn14 in migration, invasion and proliferation was described in PC-3 cells [35].

We now explore the manipulation of the cell culture conditions as a potential tool to turn the TWEAK/Fn14 system against the tumor. We report that the inflammatory cytokines and survival factor from serum modulate the response of PC-3 cells to TWEAK. In the absence of serum TWEAK/Fn14 pathway activation promotes apoptosis in androgen-independent PC-3 cells, but not in androgen-sensitive LNCaP cells. A better understanding of this regulation may turn a potential advantage of tumor cells into a therapeutic opportunity.

**Figure 1:**
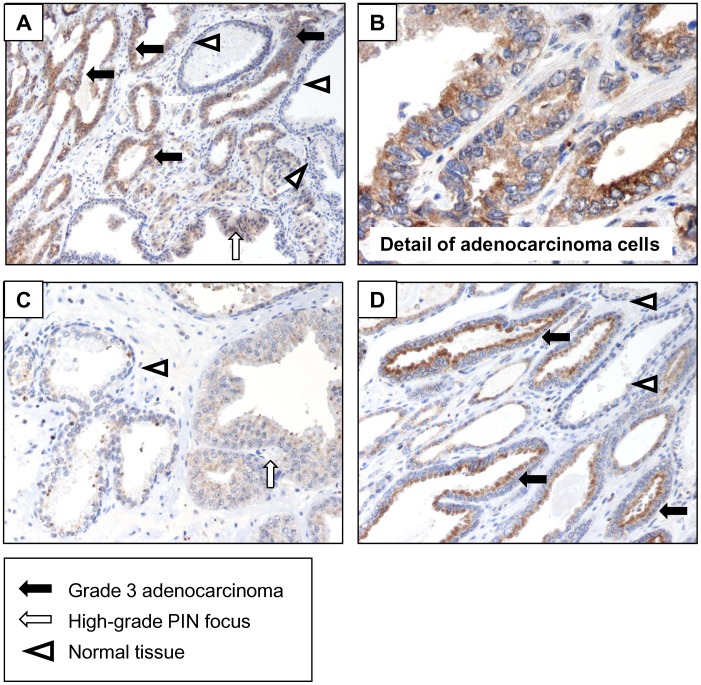
Fn14 expression in human prostate carcinoma. A) Prostate adenocarcinoma Gleason score 7 (3+4). Original magnification×200. Fn14 staining is very positive in grade 3 adenocarcinoma (black arrows) and mildly positive in a high-grade PIN focus (white arrow). No or little staining is observed in normal gland (arrowheads). **B)** Detail of adenocarcinoma cells from the same biopsy. Original magnification×400. **C)** Prostate adenocarcinoma Gleason score 6 (3+3). Mild Fn14 staining in high-grade grade PIN focus (white arrow), while normal tissue is negative (arrowhead). Original magnification×200. **D)** Fn14 staining is very positive in grade 3 adenocarcinoma (black arrows), while normal tissue is negative (arrowheads). Original magnification×200.

## Methods

### Cells and Reagents

Two human prostate cancer cell lines were used: the androgen-independent PC-3 cell line and androgen-sensitive LNCaP cells (ATCC, Rockville, MD; CRL 1435 and 1740, respectively) [36], [37]. Cells were grown in RPMI 1640 with 10% fetal bovine serum (FBS), 2 mM glutamine, and antibiotics (100 U/ml of penicillin and 100 µg/ml of streptomycin), in 5% CO2 at 37°C. RPMI-1640, penicillin, streptomycin, and trypsin-EDTA were from BioWhittaker (Waltham, MA) and FBS from Gibco (Carlsbad, CA). For experiments, cells were serum-depleted for 24 hours, and then treated with different stimuli. As positive control of Fn14 expression human proximal tubular epithelial (HK-2) cells (ATCC, CRL 2190) were used [38].

**Figure 2:**
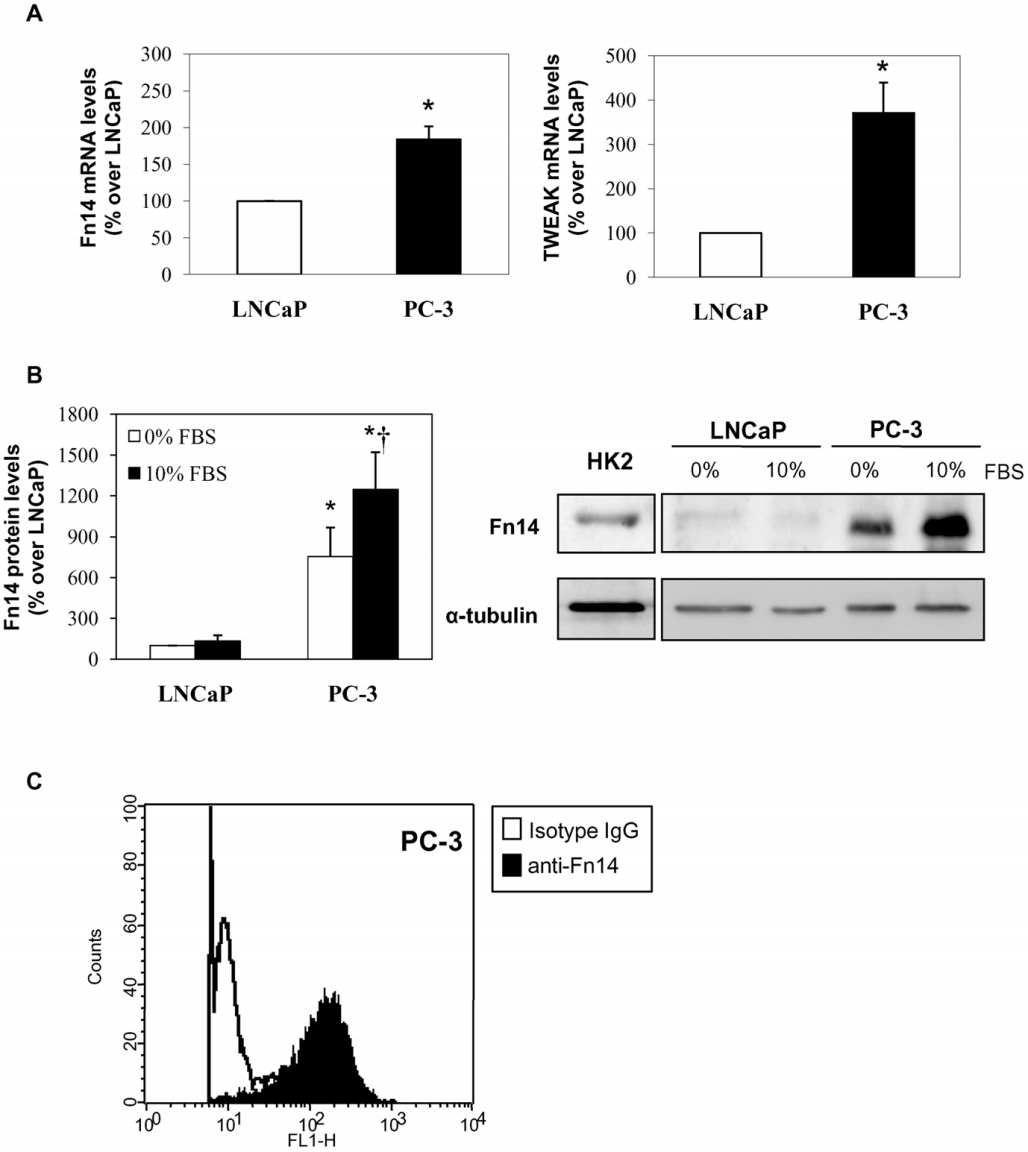
Fn14 expression in human prostate cancer PC-3 and LNCaP cell lines. **A)** Analysis of Fn14 and TWEAK mRNA expression by quantitative RT-PCR. Mean (±SEM) of three independent experiments; *p<0.05 vs LNCaP cells. **B)** Analysis of Fn14 protein expression by western blot in human prostate cancer cells cultured with or without 10% FBS for 24 hours. Mean (±SEM) of three independent experiments.*p<0.02 vs LnCAP cells. ^†^p<0.05 vs 0% FBS PC-3 cells. HK2 cells are shown as positive control**. C)** Analysis of cell surface expression of Fn14 by flow cytometry. Representative experiment. Cells were stained with an isotype-matched antibody (white area) or anti-Fn14 antibody (black area).

Recombinant human TWEAK (Millipore, Billerica, MA), unless otherwise specified, was used at 100 ng/mL. ITEM-2 neutralizing anti-Fn14 antibody (eBioscience, San Diego, CA), neutralizing anti-TWEAK antibody (BioLegend, San Diego, CA) and neutralizing anti-TNF antibody (BioLegend) were added 1 hour prior to stimuli. Human TNFα (PrePotech, London, UK) at 30 ng/mL, and human interferon-γ (IFNγ) (PrePotech) at 30 U/ml, were used in some experiments. All these concentrations are based on prior dose-response studies performed in our lab [33], [34]. Benzyloxycarbonyl-Val-Ala-DL-Asp-fluoromethylketone (zVAD-fmk) (Calbiochem, San Diego, CA, USA) was dissolved in DMSO and added 1 hour prior to stimuli. Final concentration of DMSO in culture did not modulate cell death. Bax inhibitor peptide, P5, was dissolved in water and was added 1 hour prior to stimuli (Tocris, Bristol, UK) [38].

**Figure 3:**
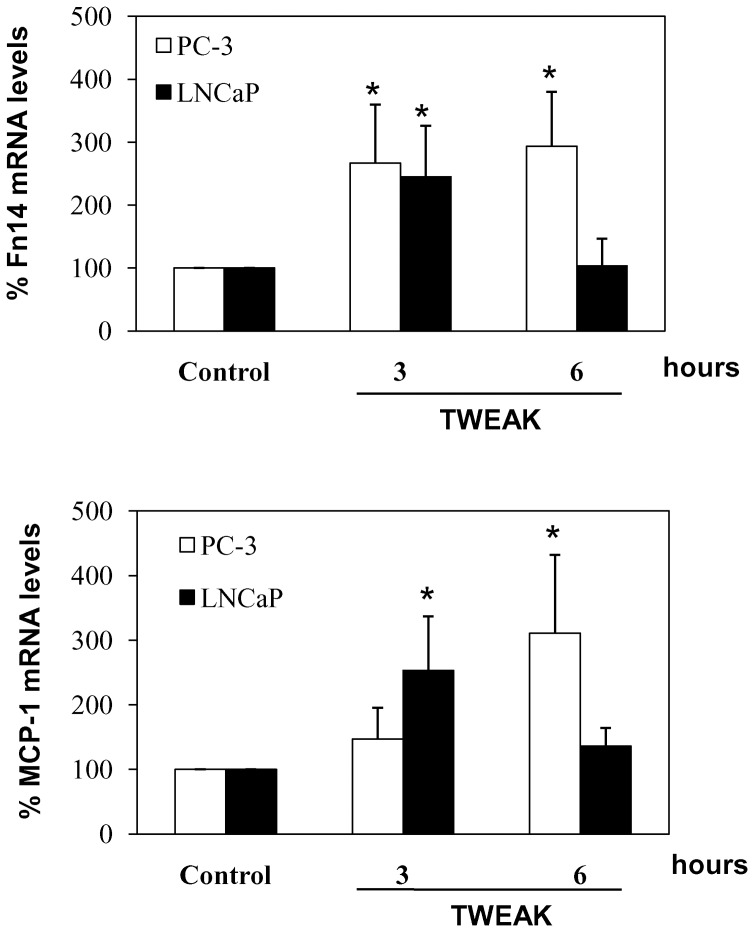
PC-3 and LNCaP cells respond to TWEAK treatment. PC3 and LNCaP cells were stimulated with 100 ng/mL TWEAK for the indicated points, and Fn14 and MCP-1 mRNA levels were measured by RT-PCR. Both cell lines respond to TWEAK, but, the PC-3 response is more sustained compared to LNCaP cells. Mean (±SEM) of three independent experiments.*p<0.05 vs control.

### Cell Surface Fn14 Expression

Cells were plated at a density of 8×10^4^ cells in twelve-well plates. Following stimulation they were detached with 2 mM EDTA/1%BSA in PBS, washed, and resuspended in PBS/1% BSA and blocked for 4 min. Then cells were incubated with 1 µg/ml anti-Fn14 ITEM4 antibody (eBioscience, San Diego, CA) or an isotype-matched control antibody for 30 min on ice. Cells were washed twice, blocked for 4 min and incubated with Alexa488-labeled goat anti-mouse IgG antibody (1/300, Invitrogen, Carlsbad, CA) for 45 min on ice in the dark. Following two additional washes with PBS/1%BSA, cells were resuspended in 1% filtered paraformaldehyde in PBS and analyzed by flow cytometry using BD CellQuest Software (BD Biosciences, San Jose, CA) [39].

**Figure 4:**
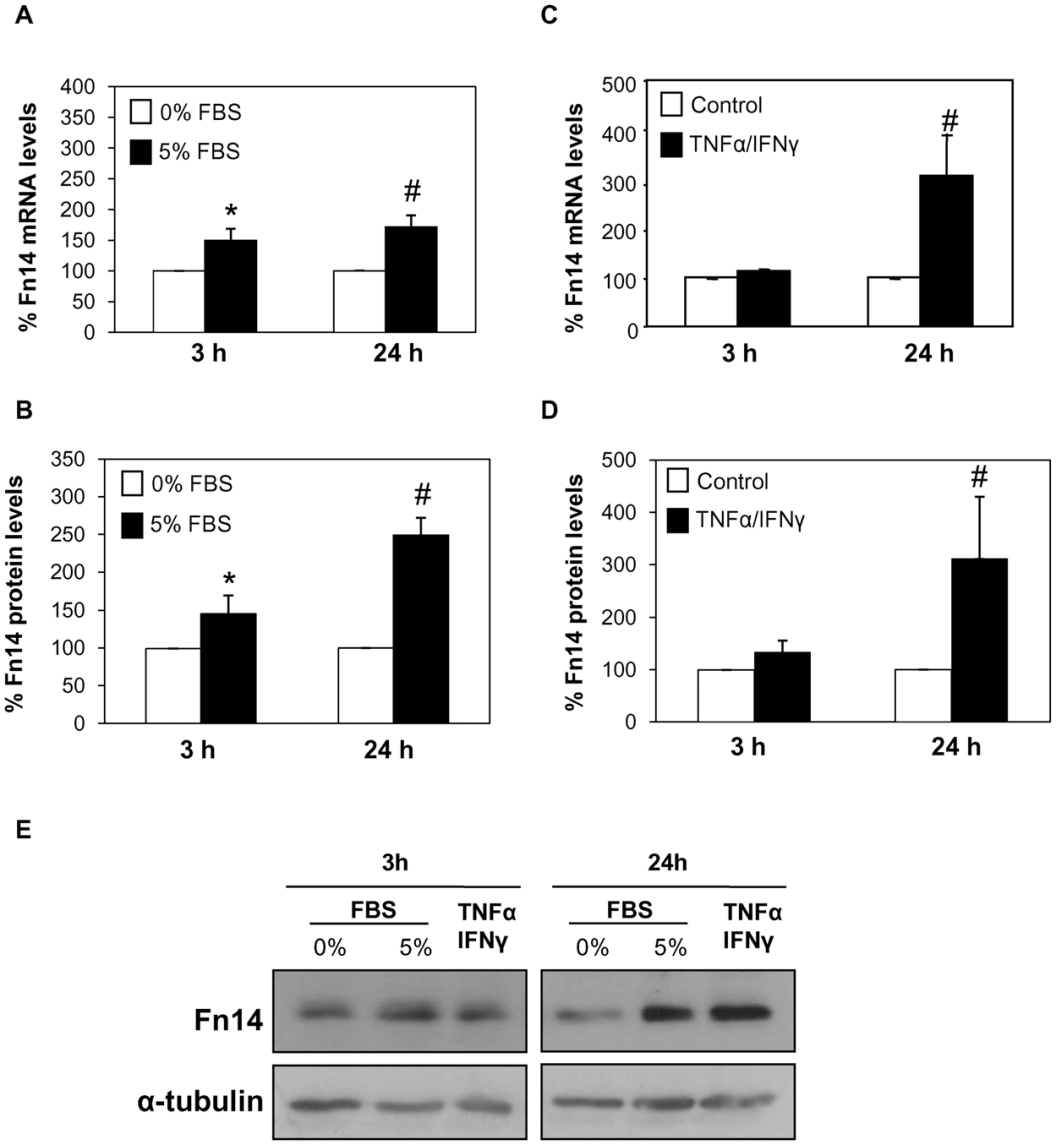
Fn14 expression in PC-3 cells is dependent on the microenvironment. A, B) PC-3 prostate cancer cells were cultured with 0% or 5% FBS for 3 or 24 hours. Fn14 mRNA expression was measured by quantitative RT-PCR **(A)** and Fn14 protein expression was studied by Western blot **(B)**. Mean (±SEM) of four independent experiments. *p<0.03 versus 0% FBS 3h; #p<0.003 vs 0% FBS 24 hours. **C, D)** Analysis of Fn14 mRNA expression **(C)**, and Fn14 protein expression **(D)** in PC-3 cells treated with TNFα (30 ng/mL)/IFNγ (30 u/mL) for 3 or 24 hours. Mean (±SEM.) of four independent experiments. #p<0.03 vs control 24 hours. **E)** Representative Western blot of Fn14 in PC-3 cells cultured with 0%FBS, 5%FBS or TNFα/IFNγ for 3 and 24 hours.

### RNA Extraction and Real-Time Polymerase Chain Reaction

Total RNA was extracted from cells by the TRI Reagent method (Sigma) and 1 µg of RNA was reverse transcribed with High Capacity cDNA Archive Kit (Applied Biosystems, Foster City, CA). Pre-developed primer and probe assays for Fn14, and GAPDH (human) were from Applied (Applied Biosystems). Quantitative PCR was performed by 7500 Real Time PCR System with the Prism 7000 System SDS Software (Applied Biosystems) and RNA expression of different genes was corrected for GAPDH [40].

**Figure 5:**
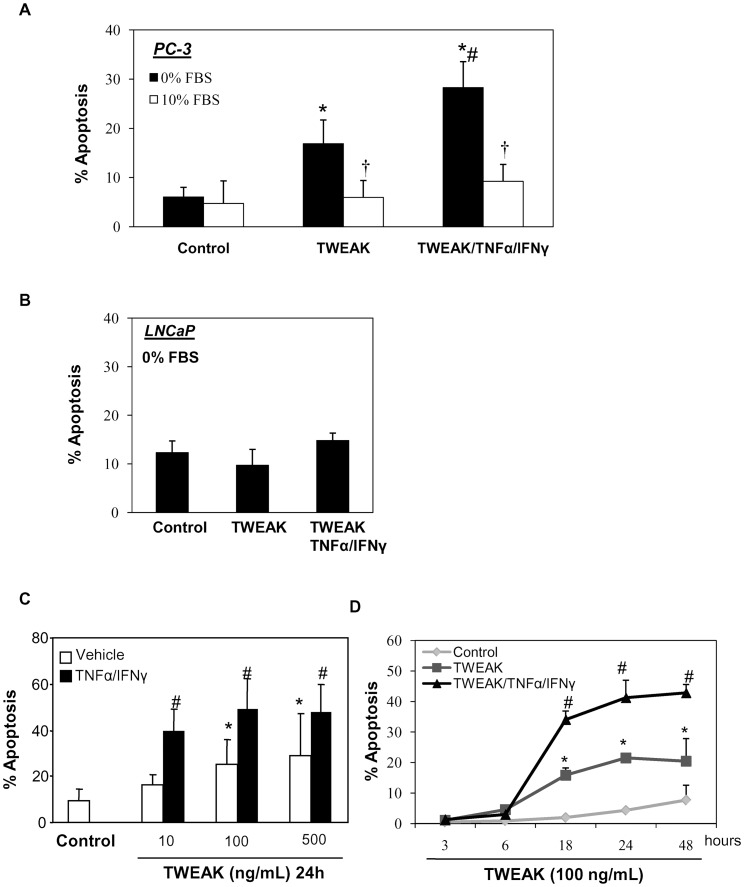
TWEAK induces apoptosis in serum depleted androgen-independent PC-3 cells but not in androgen-sensitive LNCaP cells. Cell death was assessed by flow cytometry of DNA content. Hypodiploid cells were considered apoptotic. A) PC-3 cells, cultured with or without 10% FBS, were stimulated with TWEAK (100 ng/mL) alone or in presence of TNFα (30 ng/mL)/IFNγ (30 U/mL) for 24 hours. Mean (±SD) of four independent experiments. *p<0.002 vs control; #p<0.001 vs TWEAK alone; †p<0.002 vs 0% FBS with stimuli. B) LNCaP cells, cultured without FBS, were stimulated with TWEAK alone or in combination with TNFα/IFNγ for 24 hours. Mean (±SD) of three independent experiments. C) TWEAK, alone or in combination with TNFα/IFNγ, induces apoptosis in PC-3 cells in a dose-dependent manner. Mean (±SD) of three independent experiments. *p<0.002 vs control; #p<0.0001 vs control. D) Time-course of TWEAK-induced apoptosis in PC-3 cells, alone or in combination with TNFα/IFNγ. Mean (±SD) of three independent experiments. *p<0.002 vs control; #p<0.0001 vs control.

### Western Blot

Cell samples were homogenized in lysis buffer (50 mM TrisHCl, 150 mM NaCl, 2 mM EDTA, 2 mM EGTA, 0.2% Triton X-100, 0.3% NP-40, 0.1 mM PMSF and 1 µg/ml pepstatin A) then separated by 12% SDS-PAGE under reducing conditions. After electrophoresis, samples were transferred to PVDF membranes (Millipore), blocked with 5% skimmed milk in PBS/0.5% v/v Tween 20 for 1 hour, washed with PBS/Tween, and incubated with rabbit polyclonal anti-Fn14 (1∶1000, Cell Signaling, Danvers, MA), mouse monoclonal anti-BclxL (1∶500, Santa Cruz Biotechnology, Santa Cruz, CA), rabbit polyclonal anti-Bax (1∶500, Santa Cruz Biotechnology), or rabbit polyclonal anti-cleaved active caspase 3 (1∶500, Cell Signaling). Anti-Fn14 was diluted in 5% BSA and the others antibodies were diluted in 5% milk PBS/Tween. Blots were washed with PBS/Tween and incubated with appropriate horseradish peroxidase-conjugated secondary antibody (1∶2000, Amersham, Aylesbury, UK). After washing with PBS/Tween blots were developed with the chemiluminescence method (ECL) (Amersham). Blots were then probed with mouse monoclonal anti-α-tubulin antibody (1∶2000, Sigma) and levels of expression were corrected for minor differences in loading [41].

**Figure 6:**
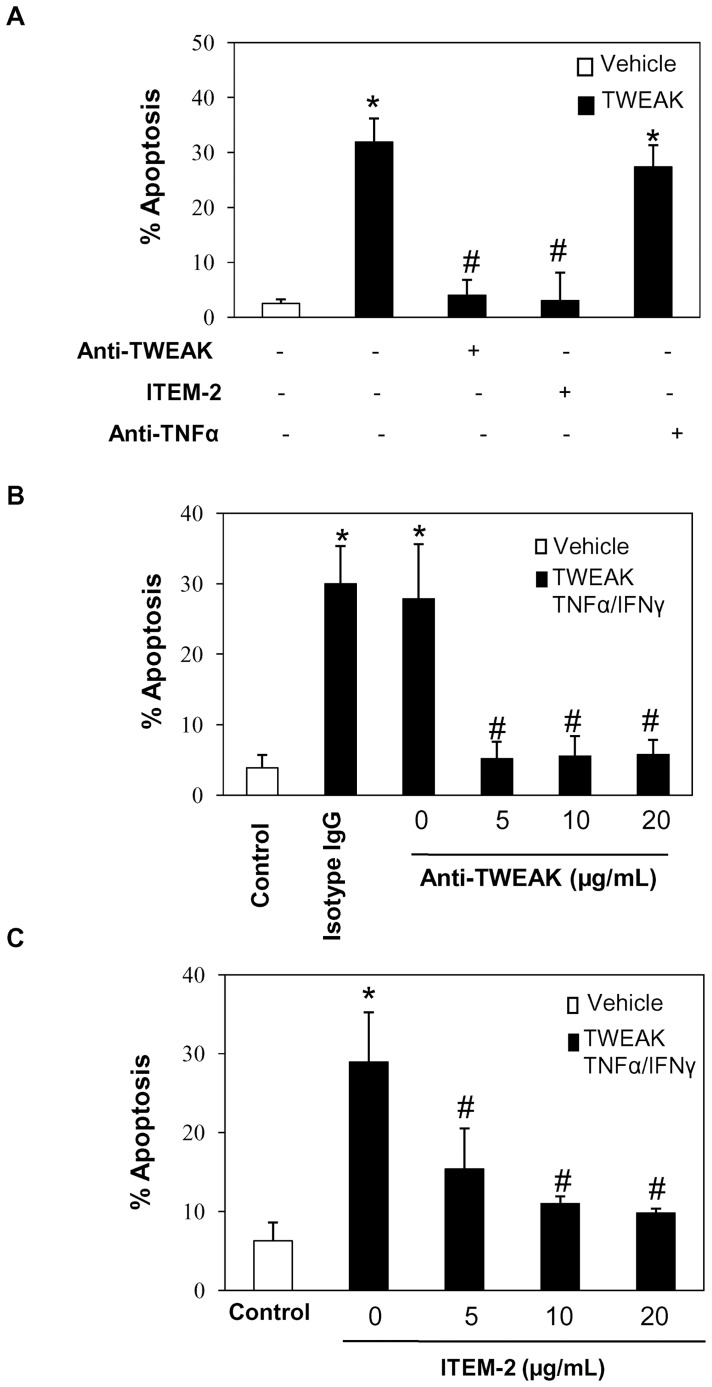
TWEAK or Fn14 antagonism prevents apoptosis induced by TWEAK or TWEAK/TNFα/IFNγ in PC-3 cells. Cell death was assessed by flow cytometry of DNA content. **A)** PC-3 cells were pre-treated with anti-TWEAK neutralizing antibody (10 µg/ml), ITEM2 anti-Fn14 antibody (10 µg/ml), or anti-TNFα neutralizing antibody (10 µg/ml), added 1 hour before TWEAK. Cell death was assessed at 24 hours. Mean (±SD) of three independent experiments. *p<0.02 vs control; #p<0.0001 vs TWEAK alone. **B), C)** PC-3 cells were pre-treated with anti-TWEAK neutralizing antibody **(B)** or with ITEM2 anti-Fn14 antibody **(C)**, added 1 hour before TWEAK/TNFα/IFNγ. Cell death was assessed at 24 hours. Mean (±SD) of three independent experiments. *p<0.02 vs control; #p<0.0001 vs TWEAK/TNFα/IFNγ alone.

### Apoptosis and Cell Death

10,000 cells were seeded in 12-well plates (Costar, Cambridge, MA) in 10% FCS RPMI overnight. In some cases they were rested in serum-free medium for 24 hour. Thereafter, stimuli were added to subconfluent cells. Apoptosis was characterized by morphologic and functional criteria. Nuclei of formalin-fixed cells were stained with DAPI (Sigma) to observe the typical morphological changes, as previously described [33], [42]. For assessment of apoptosis by flow cytometry adherent cells were pooled with spontaneously detached cells, and incubated in 100 µg/mL propidium iodide (PI), 0.05% NP-40, 10 µg/mL RNAse A in PBS at 4°C for >1 h. This assay permeabilizes the cells, thus PI stains both live and dead cells. The percentage of apoptotic cells with decreased DNA staining (hypodiploid cells) was counted by flow cytometry using BD CellQuest Software (BD Biosciences) [33], [34], [42].

**Figure 7:**
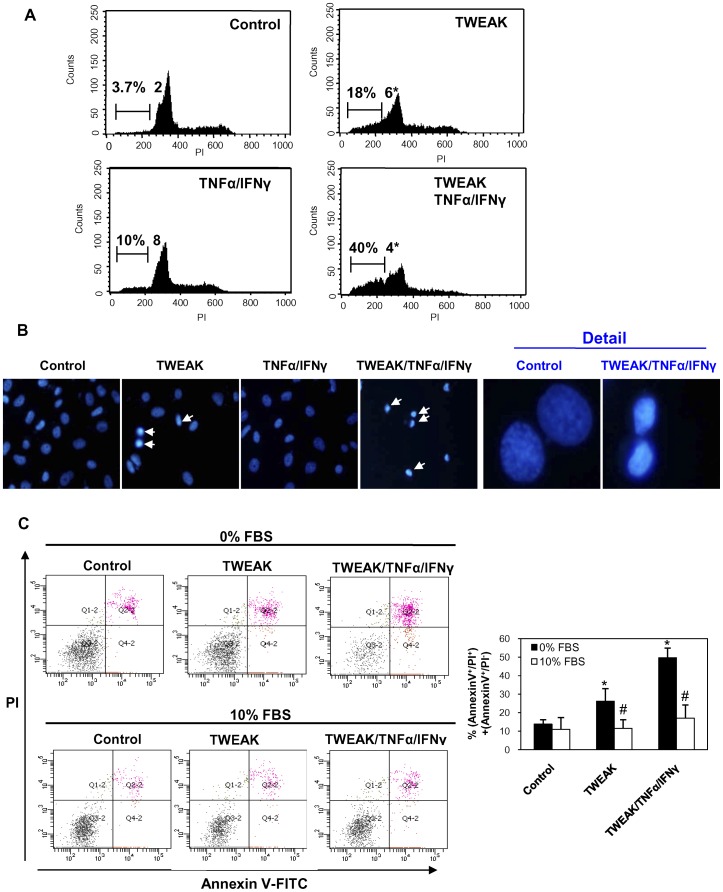
TWEAK-induced PC-3 cell death has features of apoptosis. A) Flow cytometry of DNA content. Note hypodiploid, apoptotic cells (|――|) among those treated with TWEAK, or with TWEAK/TNFα/IFNγ for 24 hrs. Mean (±SD) of four experiments.*p<0.05 vs control. **B)** Characteristic shrunk, pyknotic, fragmented nuclei are present among DAPI-stained, TWEAK or TWEAK/TNFα/IFNγ-treated cells (arrows), but are infrequent among control or TNFα/IFNγ-treated cells. Magnification x400, detail x1000. **C)** PC-3 cells were cultured with or without serum and treated with TWEAK or TWEAK/TNFα/IFNγ for 24 hours. Cells were staining with Annexin V/PI and analyzed by flow cytometry. TWEAK increases both early (AnnexinV^+^/PI^−^) and late (Annexin V^+^/PI^+^) apoptosis under serum deprivation conditions. Graphic shows percentage of early and late apoptosis [(AnnexinV^+^/PI^−^)+(AnnexinV^+^/PI^+^)]. Mean (±SD) of three independent experiments. *p<0.008 vs control; #p<0.001 vs 0% FBS with stimuli.

To quantify cell death, cells were resuspended in 100 µl of PBS and stained with 100 µg/mL PI just before to flow cytometry. This assay is based on the known ability of PI to enter in dead cells. The percentage of dead cells (stained with PI) and live cells (not stained cells) was counted by flow cytometry using BD FACS Diva Software (BD Biosciences).

**Figure 8:**
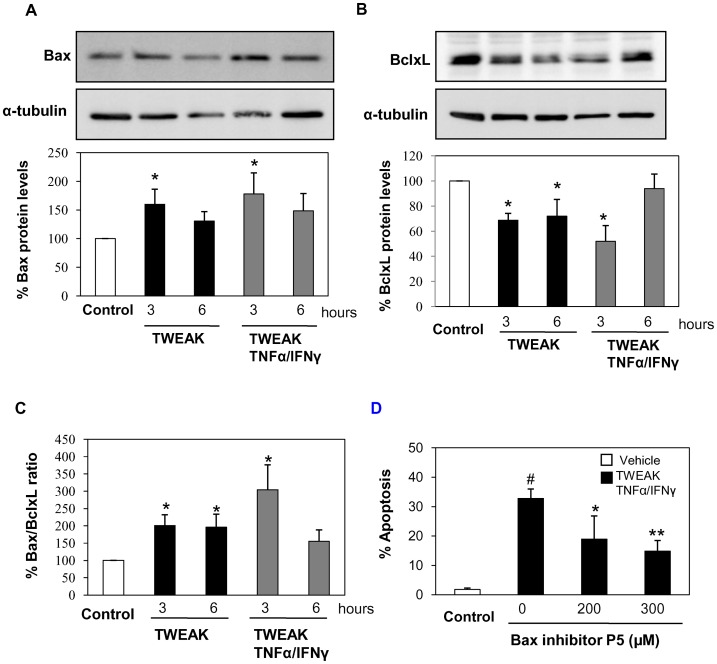
TWEAK modulates Bax and BclxL protein levels in PC-3 cells. A), B) PC-3 cells were stimulated with TWEAK or TWEAK/TNFα/IFNγ, and Bax (**A**) and (**B**) BclxL protein levels were analyzed by western blot. Mean (±SEM) of three experiments.*p<0.05 vs control. **C)** Ratio of Bax/BclxL protein levels in PC-3 cells. Mean (±SEM) of three independent experiments.*p<0.05 vs control. **D)** PC-3 cells were treated with Bax inhibitor peptide P5 at indicated doses one hour before to add TWEAK/TNFα/IFNγ. Cell death was measured by flow cytometry of DNA content. Mean (±SD) of three independent experiments. #p<0.002 vs Control; *p<0.05 vs TWEAK/TNFα/IFNγ; **p<0.005 vs TWEAK/TNFα/IFNγ.

**Figure 9:**
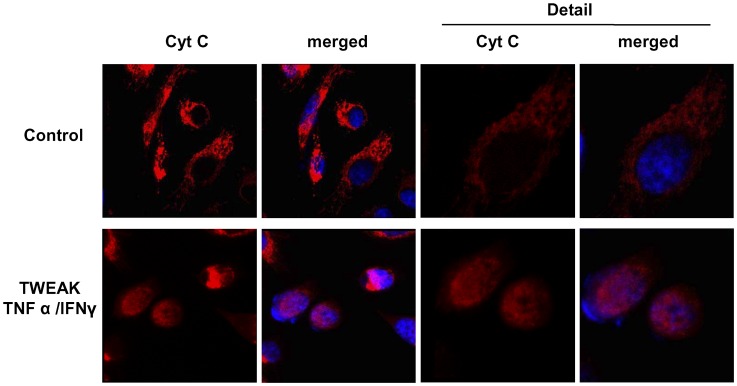
TWEAK induces cytochrome C release from the mitochondria in PC-3 cells. PC-3 cells treated with for 24 hours showed mitochondrial cytochrome C release. Confocal microscopy. Cytochrome C in red and DAPI in blue. (Magnification x400, detail x1000). Pictures representatives of three experiments.

### Assessment of Apoptosis by Annexin V-FITC

Briefly, 5×10^5^ cells were washed with ice-cold PBS, resuspended in 100 µl binding buffer, and, stained with 2.5 µl of FITC-Annexin V (Myltenyi Biotec, Bergisch Gladbach, Germany). The cells were incubated for 15 min at 37°C in the dark. Then, 200 µl of binding buffer containing PI (20 µg/mL) was added just before flow cytometry. The cells were analyzed using FACS Canto cytometer and FACS Diva Software (BD Biosciences). Early and late apoptosis was evaluated on PE fluorescence (PE for PI) versus FITC fluorescence (FITC for Annexin) plots. Cells stained with only Annexin V were evaluated as being in early apoptosis; cells stained with both Annexin V and PI were evaluated as being in late apoptosis stage.

**Figure 10:**
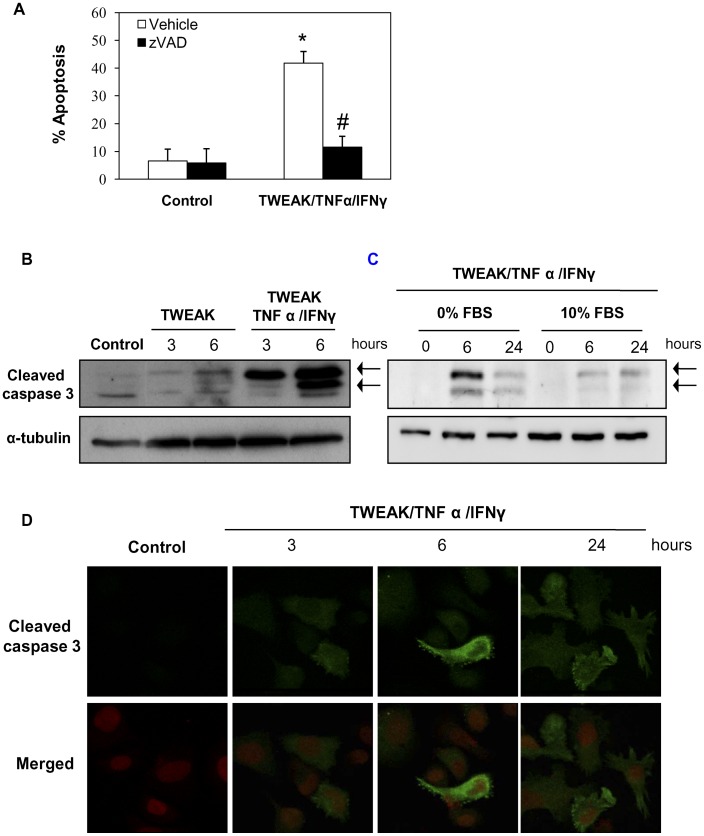
TWEAK-induced apoptosis in PC-3 cells is dependent on caspase activation. A) Inhibition of caspases with pan-caspase inhibitor zVAD (20 µM) prevented apoptosis in PC-3 cells stimulated with TWEAK/TNFα/IFNγ for 24 hours. Apoptosis was assessed by flow cytometry of DNA content. Mean (±SD) of three experiments. *p<0.002 vs control; #p<0.001 vs TWEAK/TNFα/IFNγ alone. **B**) Incubation with TWEAK or TWEAK/TNFα/IFNγ resulted in the appearance of active caspase-3 fragments (arrows, representative Western blot), **C)** and this is barely observed in presence of 10% FBS. **D)** Active **c**aspase 3 immunofluorescence in PC-3 cells treated with TWEAK/TNFα/IFNγ. Confocal microscopy. Active caspase 3 in green and propidium iodide in red. (Magnification 320x). Pictures representative of three independent experiments.

**Figure 11:**
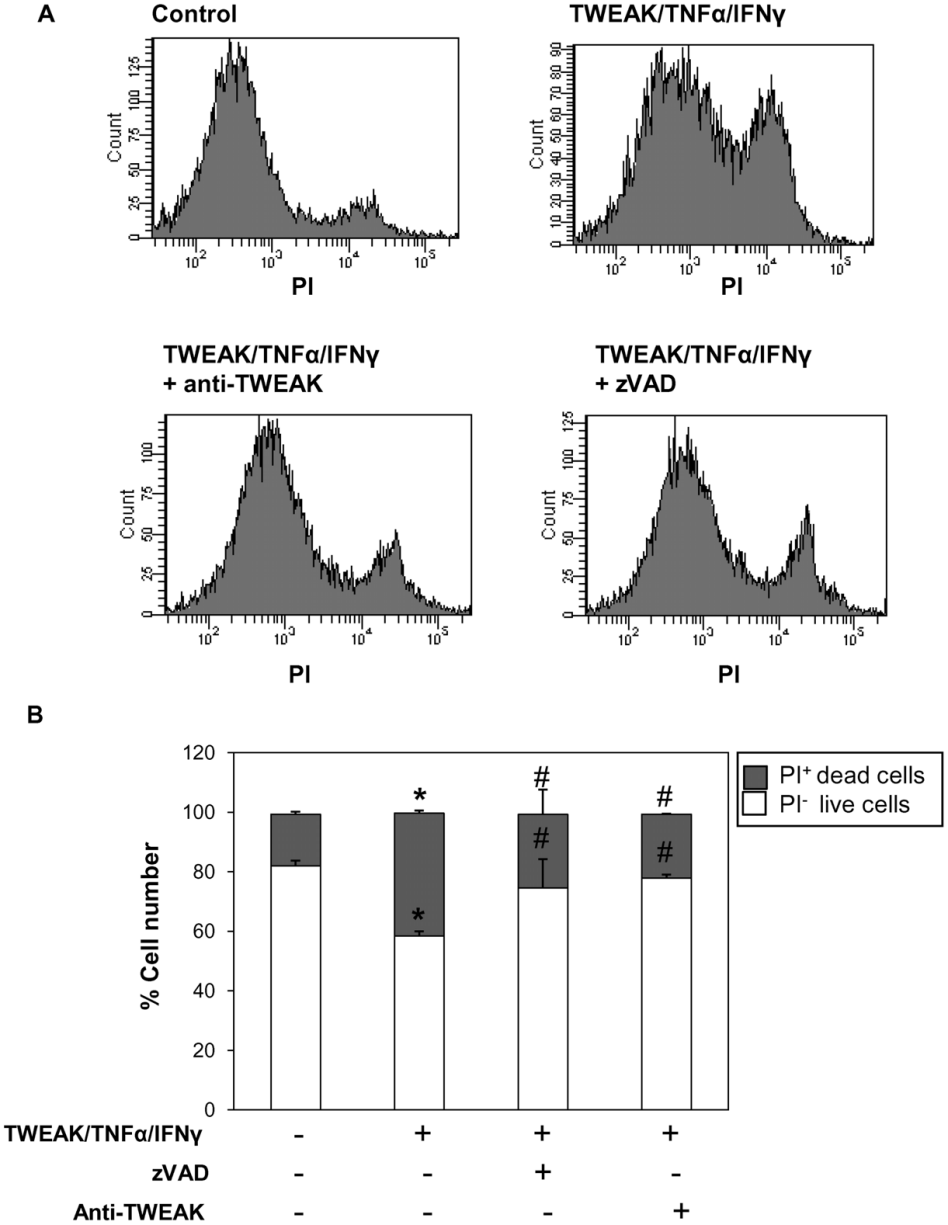
Inhibition of caspases did not induce necrotic cell death in PC-3 cells. Cell death was assessed by flow cytometry of PI staining. PC-3 cells treated with TWEAK/TNFα/IFNγ for 24 hours showed high levels of cell death, and this was significantly reduced with anti-TWEAK neutralizing antibody or with pan-caspase inhibitor zVAD. PI stains late apoptotic and necrotic cells. Mean (±SD) of three independent experiments. *p<0.005 vs control; #p<0.02 vs TWEAK/TNFα/IFNγ alone.

### Immunostaining

Cells plated onto Labtek™ slides were fixed in 4% paraformaldehyde and permeabilized in 0.2% Triton X-100/PBS, washed in PBS and incubated with rabbit polyclonal anti-active caspase 3 (1∶100, Cell Signaling) or anti-cytochrome C (1∶500, BDPharmigen) followed by Alexa-488 or Alexa-633 conjugated secondary antibody respectively (1∶300, Invitrogen). Nuclei were counterstained with propidium iodide or DAPI respectively.

### Immunohistochemistry

Fn14 immunohistochemistry was performed in 5 biopsies from patients diagnosed of prostate adenocarcinoma, aged 65 to 77 years. The Ethics Committee of Fundacion Jimenez Diaz approved the study, and informed consent was obtained from each subject. The antigenic epitope retrieval was performed in 3 µm thick sections of paraffin-embedded tissue using a PTlink device (with a high pH solution, 95°C, 20 min). The tissue slides were incubated for 30 min at room temperature with the primary antibody, rabbit polyclonal anti-Fn14 (1∶100, Cell Signaling). For immunohistochemical staining EnvisionFLEX + visualization system was used, in a DAKO Autostainerplus platform. The tissue sections were subsequently counterstained with hematoxylin. Same sections were incubated without the primary antibody as negative controls.

### Statistics

Statistical analysis was performed using SPSS 11.0 statistical software. Results are expressed as mean ±SEM for protein and mRNA expression experiments and as mean± SD for flow cytometry experiments. Significance at the p<0.05 level was assessed by Student´s t test for two groups of data and ANOVA for three of more groups.

## Results

### Expression of Fn14 in Prostate Cancer

Prostate adenocarcinoma biopsies displayed a similar pattern of Fn14 expression (**Figure 1**). Fn14 expression was negative in normal prostate epithelium, mildly positive in high-grade prostatic intraepithelial neoplasia (PIN) foci and very positive in prostate adenocarcinoma. This suggests that the cell culture observation that prostate carcinoma cell lines express Fn14 is clinically relevant and is concordant with prior reports [35].

### Constitutive Fn14 and TWEAK Expression in Human Prostate Cancer Cells

First, we studied the expression of TWEAK and Fn14, the TWEAK receptor, in two different human prostate cancer cell lines, PC-3 and LNCaP. PC-3 is an androgen-independent cell line, whereas LNCaP is an androgen-sensitive cell line. Both cell lines constitutively expressed Fn14 at the mRNA (**Figure 2** **.A**) and protein levels (**Figure 2** **.B**). Although, basal Fn14 expression, either mRNA levels or protein levels, was significantly higher in PC-3 cells compared to LNCaP cells. Moreover, basal levels of TWEAK also were higher in PC-3 cells compared to LNCaP cells, as assessed by RT-PCR (**Figure 2** **.A**).

Fn14 expression was clearly increased by fetal bovine serum (FBS) in PC3 cells, and, weakly in LNCaP cells (**Figure 2** **.B**). These results are similar to those reported by Huang et al [35], and, suggest that Fn14 may have a role in prostate cancer because is highly expressed in the more aggressive malignant cells. Finally, we demonstrated by flow cytometry that PC-3 cells expressed Fn14 in cell surface (**Figure 2** **.C**).

### TWEAK Increased Fn14 and MCP-1 Expression in Prostate Cancer Cells

TWEAK is a multifunctional cytokine that can induce inflammatory molecule production in numerous cell types [40]. We stimulated prostate cancer cells with TWEAK and measured Fn14 and MCP-1 mRNA levels by RT-PCR. We observed that TWEAK increased Fn14 and MCP-1 expression in both cell lines, demonstrating that, the two cell types have functional TWEAK receptor **(** **Figure 3** **).** However, the time course differed between the two cell lines, being more transient in LNCaP cells that in PC-3 cells.

### Regulation of Fn14 Expression in PC-3 Cells

In various cell types Fn14 expression is dependent on the microenvironment [33], [34], [43], [44]. FBS, which is rich in growth and survival factors, increased Fn14 expression in PC-3 cells at the mRNA, as assessed by RT-PCR **(** **Figure 4** **.A)** and protein levels, as measured by western blot (**Figure 4** **.B, E**).

The inflammatory cytokines TNFα and IFNγ also upregulated Fn14 mRNA (**Figure 4** **.C)** and protein (**Figure 4** **.D, E)** expression in PC-3 cells. The time course of Fn14 upregulation in response to TNFα/IFNγ was delayed with respect to observations in other cell types [33].

### Lethal Effect of TWEAK in Human Prostate Cancer Cells

TWEAK can induce apoptosis, survival or even proliferation in different tumor cell lines and other cells [27], [33], [34]. We studied the lethal effect of TWEAK over prostate cancer cell lines. TWEAK (100 ng/mL) induced apoptosis in PC-3 cultured in the absence of FBS. The lethal effect was prevented by FBS (**Figure 5** **.A**). In some cases, TWEAK-induced cell death requires co-incubation with sensitizers, such as the inflammatory cytokines TNFα and IFNγ [19], [33]. In this regard, co-incubation of TWEAK with TNFα/IFNγ strongly increased apoptosis in PC-3 cells (**Figure 5** **.A**). Neither TNFα nor IFNγ, alone or together, induced apoptosis in PC-3 cells (data not shown). However, TWEAK did not induce apoptosis in serum depleted LNCaP cells, neither alone, nor in combination with TNFα/IFNγ (**Figure 5** **.B**). These results are concordant with the effect of TWEAK observed in renal tubular cells, where, TWEAK induces proliferation in non-stressed cells, but, induces apoptosis in the presence of inflammatory cytokines [33], [34]. TWEAK-induced apoptosis in PC-3 cells is TWEAK dose-dependent (**Figure 5** **.C**) and is observed from 18 hours (**Figure 5** **.D**).

### Fn14 Mediates the Lethal Effect of TWEAK Over PC-3 Cells

While Fn14 is the only characterized TWEAK receptor, there is evidence for a second TWEAK receptor, and TWEAK can bind to CD163 [45], [46]. Therefore, we investigated whether TWEAK induces apoptosis in PC-3 cells through Fn14 activation. Pre-treatment with neutralizing anti-Fn14 (ITEM-2) or anti-TWEAK antibodies prevented TWEAK- (**Figure 6** **.A**) or TWEAK/TNFα/IFNγ-induced apoptosis (**Figure 6** **.B-C**). These results suggest that TWEAK induces apoptosis in PC-3 cells through Fn14 activation. However, TWEAK may induce apoptosis through recruitment of endogenous TNF/TNF receptor 1 (TNFR1) [47]. To rule out this mechanism, we pre-stimulated PC-3 cells with an anti-TNF neutralizing antibody. Anti-TNF treatment did not prevent TWEAK-induced apoptosis in PC-3 cells (**Figure 6** **.A**).

### Characterization of TWEAK-induced Apoptosis in PC-3 Cells

TWEAK-induced apoptosis in PC-3 cells was assessed both by the presence of hypodiploid cells measured by flow cytometry and by the typical morphology (nuclear shrinkage, condensation and fragmentation as well as decreased cell size) (**Figure 7** **.A–B**). In Annexin V/PI assays, TWEAK increased the percentage of apoptotic cells in PC-3 cells cultured the in absence of serum, and this was more evident in cells treated with TWEAK/TNFα/IFNγ. However, neither TWEAK nor TWEAK/TNFα/IFNγ increased the number of apoptotic cells in the presence of serum (**Figure 7** **.C**). In addition, TWEAK did not increase Annexin V/PI positive cells in the LNCaP cell line (data not shown). We then studied the levels of proteins of the Bcl2 family. TWEAK and TWEAK/TNFα/IFNγ increased proapoptotic Bax levels (**Figure 8** **.A**), downregulated antiapoptotic BclxL levels (**Figure 8** **.B**), and increased the final Bax/BclxL ratio (**Figure 8** **.C**). These results indicate that TWEAK modulates proteins of the Bcl2 family to favor the apoptosis cell death. Moreover, a Bax inhibitor peptide (P5) dose-dependently decreased TWEAK-induced apoptosis in PC3 cells, further suggesting recruitment of Bax and the mitochondrial pathway (**Figure 8** **.D**). To confirm the involvement of the mitochondrial apoptosis pathway we performed immunofluorescence of cytochrome C (Cyt C). Unstimulated cells showed mitochondrial Cyt C staining, whereas, in presence of TWEAK/TNFα/IFNγ some cells showed Cyt C release, indicating that this pathway is activated (**Figure 9**).

### TWEAK-induced Apoptosis in PC-3 Cells is Caspase Dependent

Next, we studied the mechanisms of TWEAK-induced apoptosis in PC-3 cells. Pretreatment with a pan-caspase inhibitor, zVAD, prevented TWEAK/TNFα/IFNγ-induced apoptosis in PC-3 cells, indicating that the apoptosis is caspases-dependent (**Figure 10** **.A**). Western blot showed processing of pro-caspase 3 to yield active caspasa-3 in presence of TWEAK at 6 hours. This effect was stronger and earlier in presence of TWEAK/TNFα/IFNγ (**Figure 10** **.B**). In presence of 10%FBS the TWEAK/TNFα/IFNγ combination barely activated caspase 3 (**Figure 10** **.C**). Immunofluorescence using an anti-active caspase-3 antibody confirmed caspase-3 activation (**Figure 10** **.D**). In some cells stimulated with TNFSF cytokines, caspases inhibition prevents apoptotic cell death, but, induces necrotic cell death [33]. In cell death assays staining with PI, zVAD protected from the cell death induced by TWEAK/TNFα/IFNγ at the same level that anti-TWEAK antibody. This result indicates that caspase inhibition does not induce necrosis in PC-3 cells stimulated with TWEAK (**Figure 11**).

## Discussion

The main finding is that cell culture conditions and, possibly the in vivo microenvironment can be manipulated to sensitize androgen-independent prostate cancer cells to TWEAK-induced apoptosis. Inflammatory cytokines and serum regulate both the expression of Fn14 in human cancer cell line PC-3, as well as the cell sensitivity to the lethal effect of TWEAK. Interestingly, the PC-3 androgen-independent cell line was sensitive to the lethal effect of TWEAK when cultured in the absence of the survival and mitogenic factors contained in serum. Moreover, an inflammatory milieu composed of the combination of TNFα/IFNγ increased the lethal effect of TWEAK over these cells. This offers new potential therapeutic opportunities for androgen-independent prostate cancer.

We studied the TWEAK/Fn14 system in androgen-sensitive LNCaP cells and in androgen-independent PC3 cells. Confirming previous reports [35] we observed a higher expression of Fn14 in PC-3 cells than in LNCaP cells. LNCaP cells expressed non-inducible, low levels of Fn14. Both LNCaP and PC-3 cells respond to the pro-inflammatory effect of TWEAK. However, the apoptotic response of both prostate cancer cell lines to TWEAK differed. LNCaP cells were resistant to TWEAK-induced apoptosis, both in the presence or absence of TNFα/IFNγ. More interesting were the results obtained in androgen-independent PC-3 cells, since androgen independent prostate cancer poses a more significant therapeutic challenge. Serum-deprived PC-3 cells were spontaneously sensitive to TWEAK-induced apoptosis and this effect was increased in an inflammatory milieu. Huang et al [35] reported that Fn14 increased proliferation and prevented basal apoptosis in PC-3 cells. However, these results were obtained in PC-3 cells cultured in the presence of FBS, and we observed the lethal effect of TWEAK over PC-3 cells cultured in the absence of serum. This phenomenon has already been observed for non-prostate cells. Thus, TWEAK alone induced proliferation, not cell death, in cultured, non-tumor, non-stressed renal tubular epithelial cells [34]. Upregulation of the Fn14 receptor by the growth factors present in serum increased the sensitivity of tubular cells to TWEAK-induced proliferation [34]. By contrast, upregulation of Fn14 expression in serum-deprived tubular cells by inflammatory cytokines (TNFα/IFNγ) changed the response to TWEAK from proliferation to cell death [33], [34].

An inflammatory milieu resulted in a sustained increase in the expression of Fn14 in PC-3 cells. However, similar to observation in tubular cells [34], the level of Fn14 expression may not be the single mechanism implicated in TWEAK sensitization, as serum also increased Fn14 expression but did not sensitize to cell death by TWEAK in PC-3 cells. A similar pattern of sensitivity to another TNFSF cytokine has been described: PC-3 were reported to be highly sensitive to TRAIL-induced apoptosis, and LNCaP to be resistant [48]. Although not confirmed by all authors [49] this observation may have a biological basis: androgens positively regulate the expression of the antiapoptotic FADD-like interleukin-1beta-converting enzyme (FLICE)-like inhibitory protein (FLIP) [50], which blocks transmission of the lethal signal from TNFRSF receptors that possess a death domain (DD) [51]. This information is pertinent despite Fn14 lacking a DD because one of the potential pathways for TWEAK-induced apoptosis is sensitization to minor amounts of other TNFSF cytokines present in the microenvironment [52].

We also characterized the molecular and cellular mechanism of the cell death induced by TWEAK in PC-3 cells. PC-3 treated with TWEAK showed characteristics of apoptosis, such as presence of hypodiploid cells, the typical morphology (nuclear shrinkage, condensation and fragmentation as well as decreased cell size) and Annexin V/PI staining. Furthermore, we observed that TWEAK modulated proteins of Bcl2 family, increasing the Bax/BclxL ratio, and a Bax inhibitor peptide dose-dependently prevented cell death. TWEAK-induced apoptosis in PC-3 cells was caspase-dependent. Different to other cell systems caspase inhibition did not sensitize PC-3 cells to necrotic death [33].

Natural sources of TWEAK in the prostate may include leukocytes. Thus, T cells express an array of lethal cytokines, such as TRAIL and TWEAK that are functional in target cell killing [53], [54]. Furthermore, TWEAK mediates the anti-tumor effect of tumor-infiltrating macrophages [55]. Since these are inflammatory cells, this information, together with our observation, suggests that TWEAK may have a role in natural defenses against prostate cancer. In addition, TWEAK circulates in serum [56]. However, circulating TWEAK levels may decrease under diverse pathological circumstances, including chronic kidney disease, vascular injury and diabetes [57]–[59]. Interestingly, diabetes appears to worsen the outcomes of prostate cancer [60], [61]. It would be worth measuring circulating TWEAK levels in metastatic prostate carcinoma, since low TWEAK availability might be one of the factors facilitating tumor expansion once it reaches an androgen-resistant stage. In addition, CD163, a scavenger TWEAK receptor expressed by certain macrophages [45] and may compromise TWEAK availability in the context of an inflamed prostate. In this regard, a biological therapy approach destined to activate the Fn14 receptor in androgen resistant prostate cancer may complement natural anti-tumor defenses. The use of an activating antibody may bypass regulatory or maladaptive mechanisms that lower TWEAK levels.

The recent availability of Fn14 targeting antibodies may enhance the spectrum of prostate tumors sensitive to TWEAK/Fn14 modulating therapies. Two different anti-Fn14 antibodies have shown anti-tumor activity in cultured cells and experimental models [26] [62], [63]. A phase 1 dose escalation trial of PDL192 humanized anti-TWEAK receptor monoclonal antibody in subjects with advanced solid tumors was recently completed [30]. An agonistic anti-Fn14 antibody undergoing clinical trials appears to have dual mechanisms of action, where binding to the target activates Fn14 and also recruits the immune system to mediate antibody-dependent cellular cytotoxicity (ADCC) activity to help destroy the tumor [26]. Recruitment of ADCC may sensitize to killing by this antibody even Fn14-expressing, TWEAK-resistant cells.

In conclusion, TWEAK activation of the Fn14 receptor induced apoptosis in androgen-independent prostate cancer cells, when stimulated with inflammatory cytokines and deprived of survival factors. Since that TWEAK/Fn14 system was previously reported to promote prostate cancer cell proliferation in the presence of serum, this information may have therapeutic consequences for treatment of androgen-independent prostate cancer by designing maneuvers that sensitize tumor cells to TWEAK-induced apoptosis or by the use of agonistic anti-Fn14 antibodies [26].
